# Modulating apical tip degeneration of the barley inflorescence: A potential target for grain yield increase

**DOI:** 10.1093/plcell/koad180

**Published:** 2023-06-26

**Authors:** Nicolas M Doll

**Affiliations:** Assistant Features Editor, The Plant Cell, American Society of Plant Biologists; Department of Plant Biotechnology and Bioinformatics, Ghent University, Ghent 9052, Belgium; VIB Center of Plant Systems Biology, Ghent 9052,Belgium

Barley is currently the fourth most-produced cereal worldwide (https://www.fao.org/faostat/). The number of grains produced from an inflorescence is an important trait for barley yield. During early development, the indeterminate barley inflorescence develops more spikelet (the basic unit of a grass flower) primordia than the final number of grains, as up to 50% of the apical spikelets die during pre-anthesis tip degeneration (PTD) (Fig.). The number of spikelet primordia affected by PTD greatly influences the final number of grains, and restricting PTD to fewer spikelets therefore represents a potential target for yield improvement in barley (Kamal et al. 2021).

**Figure.**
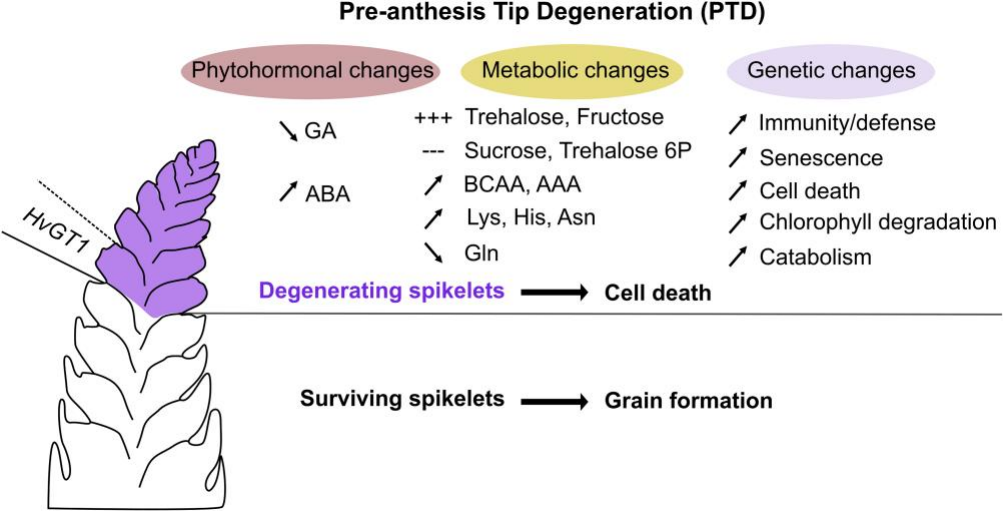
Molecular changes occurring during PTD. The tip of a young barley inflorescence is represented on the left: in the apical part the spikelet primordia that undergo PTD and eventually die, and in white, the surviving spikelets that eventually produce a grain. HvGT1 expands PTD to more basal spikelets. Figure made by N.M. Doll with Inkscape.

A careful analysis of early inflorescence development by **Nandhakumar Shanmugaraj and colleagues (Shanmugaraj et al. 2023)** shows that a consistent number of spikelets undergo PTD with conserved timing, strongly suggesting that PTD is developmentally programmed. To find potential PTD regulators, Shanmugaraj and coauthors analyzed the distribution and dynamics of the main known regulators of plant development, mostly phytohormones, metabolites, and genes within the young barley inflorescence, in both the 2-rowed cultivar Bowman and 6-rowed cultivar Morex (Fig.).

Quantification of phytohormone levels revealed different dynamics within the inflorescence: PTD was associated with a strong decrease in gibberellins and a strong increase in abscisic acid levels, specifically during later stages, while the surviving spikelets displayed high levels of cytokinins and auxin. Significant differences in metabolite distribution were also observed between the apical dying part and the surviving part of the inflorescence. Notably, they found the apical tip was depleted in sucrose and trehalose 6P but showed high levels of trehalose and fructose, a common feature in senescing organs. PTD is also associated with an accumulation of aromatic amino acids (tryptophan, tyrosine, phenylalanine), branched-chain amino acids (leucine, isoleucine), and proline, lysine, and asparagine, which may act as precursors for stress-related secondary metabolites or as alternative substrates for energy production. Conversely, Glu, the main component of plant nitrogen metabolism, is at a low concentration in the apical tip, suggesting reduced nitrogen metabolism during PTD.

The authors assessed differential gene expression by RNA-seq between the dying and nondying parts of the inflorescence at different time points. Many genes upregulated during PTD are associated with senescence; a weighted gene coexpression network analysis identified *HvS40-like*, a gene involved in barley leaf senescence (Jehanzeb et al. 2017), as a central element of the PTD gene regulatory module. PTD is also marked by the upregulation of many genes involved in cell death, chlorophyll degradation, and catabolism, 3 processes related to senescence, as well as in immunity and defense. Shanmugaraj et al. propose that a complex gene regulatory network, which involves immunity/defense, senescence, and light signaling pathways, modulates PTD.

To find key genetic regulators of PTD, the authors analyzed the expression of transcription factors in the young inflorescence and identified *GRASSY TILLERS 1* (*HvGT1*) as 1 of the top 50 most apically specific. *HvGT1* orthologs in Arabidopsis repress axillary bud growth by abscisic acid biosynthesis and sugar depletion, while the *GT1* ortholog in maize suppresses carpel formation in flowers, making HvGT1 a good candidate for suppressing apical spikelet growth and regulating PTD (Whipple et al. 2011; González-Grandío et al. 2017). Interestingly, the *hvgt1* mutant displays delayed PTD and an increased number of surviving spikelets, demonstrating that *HvGT1* is a key regulator of PTD (Fig.). Furthermore, by nucleotide polymorphism analysis among diverse 6-rowed barley accessions, the authors identified a region 8 kb upstream of *HvGT1*, whose mutations correlate with variation in the percentage of spikelet survival, paving the way for identifying other PTD regulators that act upstream of *HvGT1*.

Overall, this study is a great example of how high-resolution metabolite analysis coupled with quantitative genetics can provide new insights into a developmental process with potential translational applications. The authors showed that the barley tip undergoes deep changes in metabolites, phytohormonal signaling, and gene expression during PTD and identified *HvGT1* as a key regulator of PTD and a potential target for future improvements in barley yield. However, the extra-surviving spikelets observed in the *hvgt1* mutant remain rudimentary and unable to produce mature grains. Overcoming this sterility will be the key to increasing the grain number produced by a barley inflorescence.
